# The HipAB Toxin–Antitoxin System Stabilizes a Composite Genomic Island in *Shewanella putrefaciens* CN-32

**DOI:** 10.3389/fmicb.2022.858857

**Published:** 2022-03-21

**Authors:** Yi Zhao, Weiquan Wang, Jianyun Yao, Xiaoxue Wang, Dong Liu, Pengxia Wang

**Affiliations:** ^1^College of Life Sciences, Hebei Normal University, Shijiazhuang, China; ^2^Key Laboratory of Tropical Marine Bio-resources and Ecology, Guangdong Key Laboratory of Marine Materia Medica, Innovation Academy of South China Sea Ecology and Environmental Engineering, South China Sea Institute of Oceanology, Chinese Academy of Sciences, Guangzhou, China; ^3^Southern Marine Science and Engineering Guangdong Laboratory (Guangzhou), Guangzhou, China; ^4^University of Chinese Academy of Sciences, Beijing, China

**Keywords:** *Shewanella putrefaciens*, mobile genetic element, stability, genomic island, toxin–antitoxin

## Abstract

Composite genomic islands (GIs) are useful models for studying GI evolution if they can revert into the previous components. In this study, CGI48—a 48,135-bp native composite GI that carries GI21, whose homologies specifically integrated in the conserved *yicC* gene—were identified in *Shewanella putrefaciens* CN-32. CGI48 was integrated into the tRNA^Trp^ gene, which is a conserved gene locus for the integration of genomic islands in *Shewanella*. Upon expressing integrase and excisionase, CGI48 and GI21 are excised from chromosomes *via* site-specific recombination. The shorter attachment sites of GI21 facilitated the capture of GI21 into CGI48. Moreover, GI21 encodes a functional HipAB toxin–antitoxin system, thus contributing to the maintenance of CGI48 in the host bacteria. This study provides new insights into GI evolution by performing the excision process of the inserting GI and improves our understanding of the maintenance mechanisms of composite GI.

## Introduction

Genomic islands (GIs) are discrete DNA segments acquired by horizontal transfer, and they always differ among closely related strains. GIs vary in size from a few to several kilobase pairs and have a mosaic structure that evolves by gene acquisition and loss (Bellanger et al., 2014). Horizontal transfer of GIs can be advantageous for the host, influencing traits, such as pathogenicity, symbiosis, metabolism, phage resistance, and fitness (Dobrindt et al., 2004; Bellanger et al., 2014). Therefore, an understanding of GI evolution is critical for understanding the acquisition of these important adaptive traits.

Composite GI formation is a special type of GI evolution in which one mobile genetic element (MGE) is inserted within another or into the attachment sites of a resident GI (tandem accretion; Bellanger et al., 2014). Many composite GIs have been found through genome comparison (Bellanger et al., 2014), such as the SGI1 variant SGI1-B2 from *Proteus mirabilis* (Lei et al., 2015), ICE*St1* and CIME302 elements of *Streptococcus thermophilus* (Burrus et al., 2000), and ICE*6013* from *Staphylococcus aureus* (Smyth and Robinson, 2009). The native composite GIs have likely undergone some complicated recombination events; therefore, it is difficult to reconstruct their precise evolutionary history. To date, the formation processes of a few native composite GIs have been determined, such as the tripartite integrative and conjugative element (ICE) assembled through recombination from two GIs with integrases and one ICE without an integrase in *Mesorhizobium ciceri* (Haskett et al., 2016), the tandem structure of GI*_prfC_* inserting in the integration site for SXT/R391 ICEs in *Pseudoalteromonas* sp. SCSIO 11900 in our previous study (Wang et al., 2017). Native composite islands that can replicate their evolutionary processes under laboratory conditions would be especially useful for improving our understanding of GI evolution. Interestingly, how composite GIs maintain structural stability should also be explored.

Toxin–antitoxin (TA) systems were originally discovered on conjugative plasmids and participated in their stable maintenance in host bacteria (Ogura and Hiraga, 1983; Roberts et al., 1994). The TA system consists of two neighboring genes, encoding a stable toxin killing the cell or inhibiting cell growth and an unstable antitoxin that masks its toxicity (Wang et al., 2021). A proposed mechanism post-segregationally killing (PSK) was established based on the differential stability of the antitoxin and toxin components. In PSK, plasmid-loss cells do not survive, so the plasmid is maintained in the population (Jurenas et al., 2022). Currently, TA systems have also been found to be ubiquitous in bacterial chromosomes and have been suggested to contribute to the maintenance of integrative MGEs. For example, the MosAT system promotes the maintenance of SXT family ICE carried by some *Vibrio cholerae* strains (Wozniak and Waldor, 2009); the ParE_SO_/CopA_SO_ system stabilizes prophage CP4So in *Shewanella oneidensis* (Yao et al., 2018). Whether the TA system also participates in the maintenance of composite GI is unknown. In this study, a new composite island CGI48 was identified and characterized from *Shewanella putrefaciens* CN32 using genome comparison and excision assay. It evolved by inserting a 21-kb genomic island GI21 into the internal region of CGI48. We further show that GI21 carries a functional HipAB toxin–antitoxin system and contributes to the maintenance of CGI48 in the bacterial host.

## Materials and Methods

### Bacterial Strains and Growth Conditions

The bacterial strains and plasmids used in this study are listed in Table 1. *Shewanella* was grown in LB medium at 30°C. *Escherichia coli* WM3064 was grown in LB medium containing 0.3 mM 2,6-diamino-pimelic acid (DAP) at 37°C. Chloramphenicol (Cm; 30 μg ml^−1^), kanamycin (50 μg ml^−1^), and ampicillin (100 μg ml^−1^) were used in *E. coli*, and chloramphenicol (10 μg ml^−1^) was used in *Shewanella*. Isopropyl-*β*-D-thiogalactopyranoside (IPTG) was used as an inducer.

**Table 1:** Strains and plasmids used in this study.

Strains/plasmids	Description^a^	Reference
*Shewanella putrefaciens* strains		
CN32	*Shewanella putrefaciens* CN32 wild type	Lab stock
Δ*hipAB*	Deletion of *hipAB* genes in CN32	This study
ΔGI21	Deletion of GI21 in CN32	This study
ΔCGI48	Deletion of CGI48 in CN32	This study
CN32 P*_int_*::*lacZ*	Integration of plasmid pHGI01 in *int* promoter to monitor the CGI48 and GI21 loss in CN32 wild type	This study
Δ*hipAB* P*_int_*::*lacZ*	Integration of plasmid pHGI01 in *int* promoter to monitor the CGI48 and GI21 loss in strain Δ*hipAB*	This study
W3-18-1	*Shewanella putrefaciens* W3-18-1 wild type	Caro-Quintero et al., 2011
ANA3	*Shewanella* sp. ANA-3 wild type	Lab stock
*Escherichia coli* strains		
WM3064	RP4(tra) in chromosome, DAP-, 37°C	Dehio and Meyer, 1997
K-12 BW25113	lacI^q^ rrnB_T14_ Δ*lacZ*_WJ16_ *hsdR*514 Δara*BAD*_AH33_ Δrha*BAD*_LD78_	Baba et al., 2006
Plasmids		
pCA24N	Cm^R^; lacI^q^, IPTG inducible expression plasmid in *E. coli*	Kitagawa et al., 2005
pHipA	Cm^R^; lacI^q^, P*_T5-lac_*::*hipA*	This study
pHipB	Cm^R^; lacI^q^, P*_T5-lac_*::*hipB*	This study
pHipAB	Cm^R^; lacI^q^, P*_T5-lac_*::*hipA-hipB*	This study
pHGECm	Cm^R^; Kan^R^; IPTG inducible expression plasmid	Wang et al., 2017
pMD19-T	Amp^R^, *E. coli* cloning vector	Invitrogen
pMD19-T-*hipAB*	Amp^R^, expressing *hipAB* with its native promoter	This study
pXis_21_	Cm^R^, expression plasmid for Xis_21_ from GI21	This study
pXis_PO1_	Cm^R^, expression plasmid for Xis_PO1_ from GI*Spu*PO1	This study
pXis_ANA3_	Cm^R^, expression plasmid for Xis_ANA3_ from GI*Ssp*ANA3	This study
pInt_48_	Cm^R^, expression plasmid for Int_48_	This study
pHGI01	Kan^R^, Integrative *lacZ* reporter plasmid	Fu et al., 2014
pInt2894	Cm^R^, expression plasmid for Sputcn32_2894	This study
pHGI01-P*_int_*	pHGI01 containing 213 bp upstream of *int* (Sputcn32_2900)	This study
pHGR01	Kan^R^, replicative *lacZ* reporter plasmid	Fu et al., 2014
pHGR01-P*_hipA_*	Fuse *hipAB* promoter from CN32 with *lacZ* in pHGR01	This study
pK18*mobsacB*-Cm	Km^R^, Cm^R^, *SacB*, and suicide plasmid used for gene knockout	Wang et al., 2015
pK18Cm-*hipAB*	pK18*mobsacB*-Cm containing the homologous arms of *hipAB*	This study

a Cm^R^, chloramphenicol resistance; Kan^R^, kanamycin resistance; and Amp^R^, ampicillin resistance.

### Construction of Plasmids

The primers used in this study are listed in Table 2. The encoding regions of *xis_21_*, *xis_PO1_*, *xis_ANA3_*, *int_48_*, and *int2894* were amplified from the original bacterial host and cloned into the *Eco*RI and *Bam*HI sites of pHGECm using T4 ligase, generating pXis_21_, pXis_PO1_, pXis_ANA3_, and pInt_48_. The encoding regions of *hipA*, *hipB*, and *hipAB* were amplified from CN32 and inserted into the *Sal*I and *Pst*I sits of pCA24N, generating pHipA, pHipB, and pHipAB. The promoter and encoding region of *hipAB* was amplified from CN32 and inserted into pMD19-T, generating pMD19-T-*hipAB*. To construct the *lacZ* reporter plasmid pHGI01-P*_int_*, the reporter region of the integrase gene *Sputcn32_2900* was amplified with the primer pair pHGI01-P*_int_*-F/−R and fused with the *lacZ* gene in pHGI01. Then, the integrative plasmid pHGI01-P*_int_* was transferred into CN32 and Δ*hipAB* by conjugation and integrated into the promoter region of *Sputcn32_2900*, generating CN32 P*_int_*::*lacZ* and Δ*hipAB* P*_int_*::*lacZ*. The primer sets mob-F/int-R and Int-F/lacZ-R were used to confirm the construct. To construct pHGR01-P*_hipA_*, the promoter of *hipAB* was amplified with primers pHGR01-P*_hipA_*-F/−R from CN32, and inserted into the promoterless-*lacZ* reporter plasmid pHGR01.

**Table 2:** Primers used in this study.

Primers	Sequence (5'-3')	Purpose
Plasmid construction		
Int48-F	CCGGAATTCATGGGTAGTATTAACTCTCG	pInt_48_
Int48-R	CGCGGATCCTTATCCTCTTAGTTTTTGGTTC	
Xis21-F	CCGGAATTCATGAACCCATCAAATCACG	pXis_21_
Xis21-R	CGCGGATCCTAATTGATACTTTCGCGG	
Int2894-F	CCGGAATTCTTGTCTAAGGACTCGACGGAG	pInt2894
Int2894-R	CGCGGATCCTTATTTGTTGTTCATCATCATTATTCC	
Xis_PO1_-F	CCGGAATTCGTGAACATGAACCCATCAAATC	pXis_PO1_
Xis_PO1_-R	CGCGGATCCCTAATTGATACTTTCGCGGTTGG	
Xis_ANA3_-F	CCGGAATTCGTGAGCATGAACTCATTAAATAAAC	pXis_ANA3_
Xis_ANA3_-R	CGCGGATCCTTAGTACTTCCCATCTTCGACTG	
hipA-SalI-F	ACGCGTCGACGAACAGTTGACCATTCAGGC	pHipA
hipA-PstI-R	TGCACTGCAGTCATACCAATCCCCAACGCG	
hipB-SalI-F	ACGCGTCGACAGTGATAAACAAACGACTAC	pHipB
hipB-PstI-R	TGCACTGCAGTCATAAAAGCCATGTGACAC	
hipA-SalI-F	ACGCGTCGACGAACAGTTGACCATTCAGGC	pHipAB
hipB-PstI-R	TGCACTGCAGTCATAAAAGCCATGTGACAC	
pHGR01-P*_hipA_*-F	CCGGAATTCACTGTAGCGCATATTTAATAA	pHGR01-P*_hipA_*
pHGR01-P*_hipA_*-R	CGCGGATCCgtaatcatggTCATGAAAGCTCCCAAAGACATTATG	
pMD19-T-*hipAB*-F	TCATAAAAGCCATGTGACAC	pMD19-T-*hipAB*
pMD19-T-*hipAB*-R	GTCACCACATTAGTCCCACT	
Construction of Δ*hipAB*		
hipAB-up-F	ACATGCATGCGAGATGAAACGCTTCAACTCG	pK18Cm-*hipAB*
hipAB-up-R	CCGGAATTCCAGTGGATAGCATTGACCAAC	
hipAB-down-F	CCGGAATTCGCTCGTAATCTAACGAGGTAAG	
hipAB-down-R	AGCGTCGACCCAGGTTACTAATTCTAGTCAC	
hipAB-wF	GTTTACATAAACCAGCAGCAC	Confirmation of Δ*hipAB*
hipAB-wR	GTCCATATTACTCACCTTAGC	
Construction of Δ*hipAB* P*_int_*::*lacZ* and CN32 P*_int_*::*lacZ*		
pHGI01-P*_int_*-F	CCGGAATTCAACGTCGAATGACGTTTTTAGCG	pHGI01-P*_int_*
pHGI01-P*_int_*-R	CGCGGATCCgtaatcatggGTAGTTAAGTCCAAAATGGTGAC	
mob-F	CAGAGCAGGATTCCCGTTGAGCA	Confirmation of Δ*hipAB* P*_int_*::*lacZ* and CN32 P*_int_*::*lacZ*
LacZ-R	TATTACGCCAGCTGGCGAAAGG	
Int-F	ATGATTAAGTGTCACTTTTCAAGG	
Int-R	CATTTGGCTGCGATTAGCTC	
Primers used in determination of the excision and circled form of CGI48 and GI21		
21F	CCAAAGCGAGGTAAGACGT	ΔGI21
21R	TCGGAGACAGCGATGTATCG	
21cirF	AGTGGGACTAATGTGGTGACTAGAATT	The circled GI21
21cirR	TGCAAGTGCATGGTTTTATGATG	
48F	CCAAGTGAACGTTTATGATCGC	ΔCGI48
48R	GGTGTGTTTTTCATCGTTATGC	
48cirF	CGAGAGTCTATTCGTAGAGAC	The circled CGI48
48cirR	AGAATATGGTCTAACCAAGC	
oF	CCGGAATTCATGATTAAGTGTCACTTTTCAAGG	*cro/cI* gene
oR	CGCGGATCCTTAGTCTGTACCTTGGATTTC	
Primers used in qPCR for CGI48 and GI21 in CN32		
q48F	GGCTCGCATATTTCTGTGCAA	Determine the excision rate of CGI48
q48R	CCTTTGAGAGTGCTTTTAGCATAATG	
q21F	TTGGCGAGTTGCTCGAAATC	Determine the excision rate of GI21
q21R	GGAACTGGGATGTGTTTTATTGC	
q48cF	CGAGAGTCTATTCGTAGAGAC	Determine the circular form of CGI48
q48cR	AGAATATGGTCTAACCAAGC	
q21cF	AGTGGGACTAATGTGGTGACTAGAATT	Determine the circular form of GI21
q21cR	TGCAAGTGCATGGTTTTATGATG	
CN32gyrB-qF	TTCGTACTTTGCTGTTGACCTTCT	Reference gene
CN32gyrB-qR	CTACGGTGCCATCCAATGCT	
Primers used in qPCR for GI*Spu*PO1 in W3-18-1		
GISpuPO1-qF	AGGTCGCCGTCTCGATTTTA	Determine the excision rate of GI*Spu*PO1
GISpuPO1-qR	TGAGTCGGAAACATCATTAGACGTT	
W3181gyrB-qF	GCTCAGCCGCCTTTGTTTAA	Reference gene
W3181gyrB-qR	CGGCTCACCCGACATACC	
Primers used in qPCR for GI*Ssp*ANA3 in ANA-3		
GISspANA3-qF	GTCGAGCTCAAAGTACTCATCGAA	Determine the excision rate of GI*Ssp*ANA3
GISspANA3-qR	GCTACAGCAGAAGCTAATCTCATTACTC	
ANA3gyrB-qF	CTGGTGAGCCTGTGCTCGAT	Reference gene
ANA3gyrB-qR	CAAGCGCCGCACCTAACTTA	

Restriction sites included in oligonucleotide sequences are underlined.

### Construction of *hipAB* Deletion Mutant in CN32

The deletion mutant Δ*hipAB* was constructed based on pK18*mobsacB*-Cm as described previously (Wang et al., 2015). Briefly, the upstream and downstream regions of *hipAB* were amplified from CN32 using the primers listed in Table 2 and inserted into pK18*mobsacB*-Cm using T4 ligase, producing pK18Cm-*hipAB*. Then, pK18Cm-*hipAB* was introduced into CN32 by conjugation. After mating, cells were spread on LB plates containing Cm to screen the single crossover mutant in which pK18Cm-*hipAB* had integrated into the CN32 genome. The mutant was then grown on LB medium without antibiotics for 8 h. To select mutants in which the second recombination had occurred, the culture was diluted, spread on LB medium containing 10% sucrose, and grown at 30°C for 24–36 h. Single colonies were transferred onto LB- and LB-containing Cm plates simultaneously, and colonies sensitive to Cm were collected and confirmed by PCR followed by DNA sequencing.

### Conjugation Assays

The plasmids in this study were transferred from *E. coli* WM3064 into *Shewanella* strains by conjugation assays as described previously (Wang et al., 2015). Briefly, equal amounts of donor and recipient cells were mixed and dropped onto LB medium containing DAP. The plates were incubated at 30°C for 6–8 h, and cells were collected from the lawn and streaked on LB medium with antibiotics to select for transconjugants.

### Reporter Activity Assay

Specific *β*-galactosidase activity was determined by monitoring the absorbance at 420 nm using the Miller assay (Miller, 1972). To determine the promoter activity of *hipAB* under overexpression of HipB and HipB-HipA, plasmids pHipB or pHipAB were transformed into the *E. coli* host carrying the reporter plasmid pHGR01-P*_hipA_*. Overnight cultures were diluted 1:100 in LB with Kan and Cm and induced with 0.1 mM IPTG at an OD_600_ of 1.0. After induction for 2 h, cells were collected to determine the *β*-galactosidase activity.

### Quantification of the Excision Rate of GI21 and CGI48

For GI21, GI*Ssp*ANA3, GI*Spu*PO1, and CGI48, *attB/gyrB* indicated the excision rate of the target GIs after excision. We conducted real-time quantitative PCR (qPCR) assays to quantify the *attB* of these GIs as previously reported methods (Burrus and Waldor, 2003; Wang et al., 2017). The primers used for the qPCR assays are listed in Table 2, and chromosomal *gyrB* was used as the reference gene. To test the regulation of Xis and Int on the excision of GI21, GI*Ssp*ANA3, GI*Spu*PO1, and CGI48, pXis_21_-, pXis_PO1_-, pXis_ANA3_-, and pInt_48_-containing strains were induced with 1.0 mM IPTG for 6 h at an OD_600_ of 0.8–1.

### Calculation of % CGI48- and GI21-Free Cells

Both CGI48 and GI21 are non-replicable, and loss of CGI48 and GI21 only occurs after their excision. Therefore, to visualize the loss of CGI48 and GI21, the wild-type and Δ*hipAB* strains carrying pXis_21_ or pInt_48_ were induced with 1 mM IPTG for 6 h to overproduce Xis_21_ (to induce GI21 excision) or Int48 (to induce CGI48 excision). Then, the cells were plated on LB plates containing X-gal to calculate the numbers of white colonies (losing CGI48 or GI21) plus blue colonies, and the white colonies were also confirmed by PCR assay.

### Plasmid Stability Assay

The contribution of HipA/HipB TA system to plasmid stability was tested as described previously (Yao et al., 2015). Overnight cultures of *E. coli* BW25113 containing plasmid pHipAB or empty vector pCA24N were grown in LB medium with Cm. Then, the preculture was used to inoculate 3 ml LB without antibiotics. Every 12 h of growth, bacterial suspensions were diluted 1,000-fold in 3 ml fresh LB medium. The cultures were serially diluted in 10-fold dilution steps from 0 to 108 h, and 10 μl was dropped on LB plates with or without Cm. The colony-forming unit (CFU) assay was conducted every 12 h for 108 h, and the number of CFUs was determined. Each experiment was performed in triplicate with two independent cultures.

## Results

### CGI48 Is a Composite Island Containing GI21

**Figure 1:**
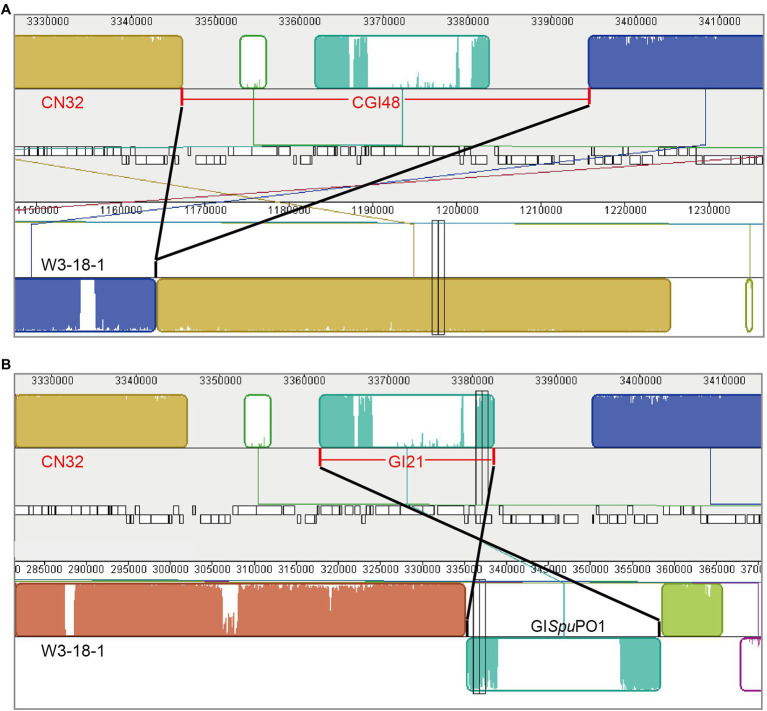
Schematic view of the composite island CGI48 in the CN32 genome. CGI48 **(A)** and its component genomic island GI21 **(B)** were identified by comparing the genome sequence of *S. putrefaciens* CN32 (CP000681) with *S. putrefaciens* W3-18-1 (CP000503) with Mauve.

**Figure 2:**
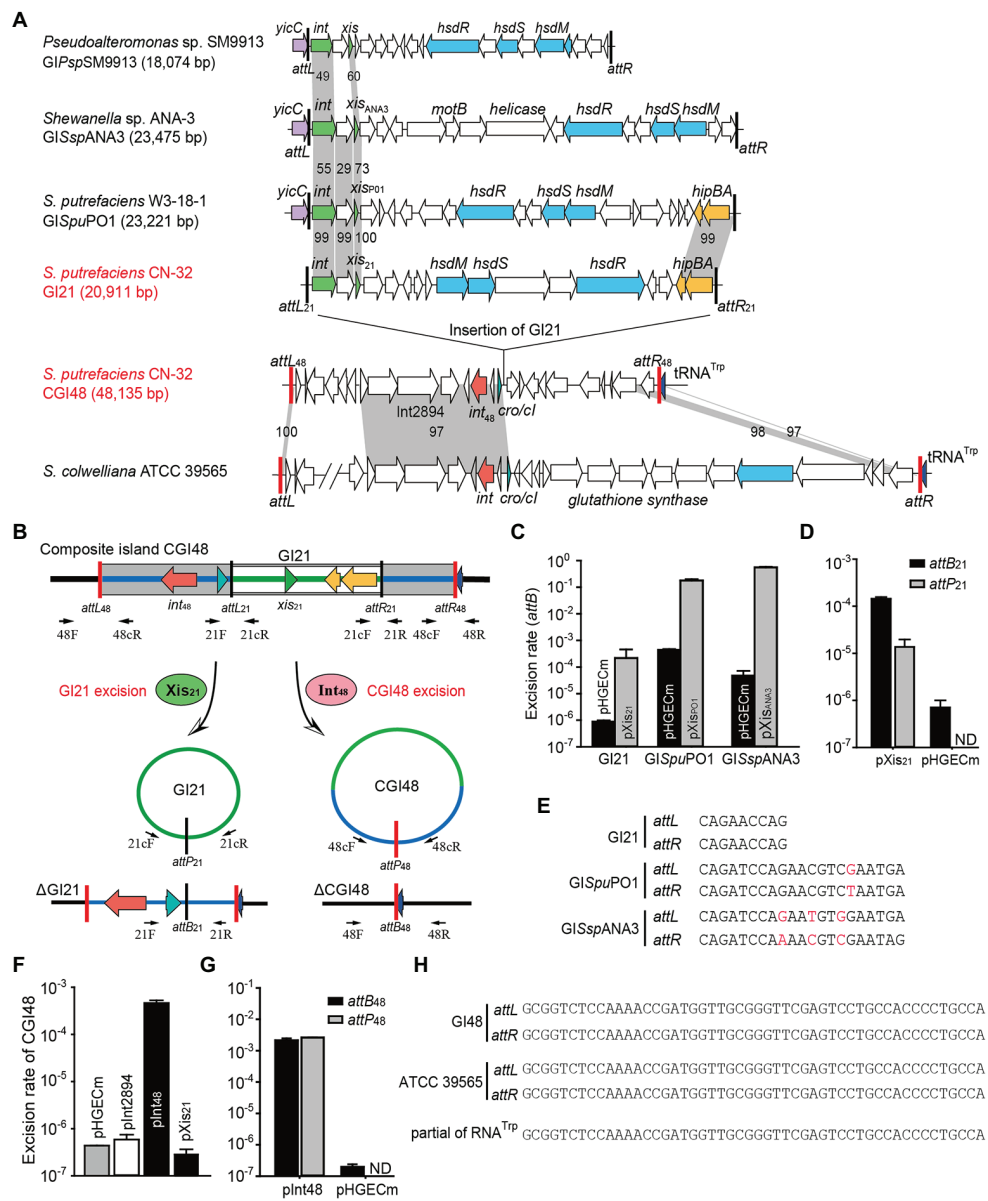
The composite island CGI48 and its component GI21 can be excised from the CN32 genome. **(A)** Sequence analysis of CGI48 with the related genomic islands. Open reading frames with putative functions are shown in different colors. The *attL* and *attR* attachment sites of CGI48 and GI21 are shown in red and black, respectively. The sequence of the GI integrated into tRNA^Trp^ of ATCC 39565 genome was in L876DRAFT_scaffold 00018.18_C (82,217–85,620 bp) to scaffold 00033.33_C (1,478–38,068 bp). **(B)** Schematic of the excision of CGI48 and GI21. **(C)** The excision rate of GI21, GI*Spu*PO1, and GI*Ssp*ANA3 was quantified when cognate excisionase was overexpressed. **(D)** Comparison of the excision rate (*attB_21_*) and circular form of GI21 (*attP_21_*) in CN32 when Xis21 was overexpressed. ND indicates not detected. **(E)** Sequence comparison of the attachments of GI21, GI*Spu*PO1, and GI*Ssp*ANA3. **(F)** The excision rate of CGI48 when Int_48_ was overexpressed. **(G)** Comparison of the excision rate (*attB_48_*) and circular form of CGI48 (*attP_48_*) in CN32 when Int48 was overexpressed. ND indicates not detected. **(H)** Sequence comparison of the attachment sites of CGI48 and GI in ATCC 39565 compared with the 3′ end of RNA^Trp^ in *Shewanella*.

Comparing the genome sequence of *S. putrefaciens* CN32 with the related strain *S. putrefaciens* W3-18-1, a large region within 3,340,000–3,400,000 of CN32 was absent in the same gene locus (1,160,000–1,170,000) of W3-18-1 (Figure 1A), suggesting that this region was acquired horizontally. Moreover, the internal sequence within 3,360,000–3,380,000 of this large region showed high homology with another region 335,000–360,000 of W3-18-1 (Figure 1B). These results suggested that the region within 3,340,000–3,400,000 of CN32 is a putative composite genomic island. It is 48 kb in length; thus, it is designated CGI48 hereafter (Table 3). Further analysis showed that region 3,360,000–3,380,000 of CN32 contains a 21 kb genomic island (designated GI21), which exhibits sequence identity with genomic islands integrated in the conserved *yicC* gene, such as GI*Spu*PO1 in *S. putrefaciens* W3-18-1, GI*Ssp*ANA3 in *Shewanella* sp. ANA-3, and GI*Psp*SM9913 in *Pseudoalteromonas* sp. SM9913 (Figure 2A). GI21 exhibits 99% sequence identity with the two ends of GI*Spu*PO1 in W3-18-1. The left region of GI21 contains an integrase and an excisionase gene next to the left attachment site (*attL*_21_), and the right region contains a putative *hipA*-*hipB* toxin–antitoxin pair next to the right attachment site (*attR*_21_). The middle region contains 12 genes encoding a restriction–modification system and hypothetical proteins (Figure 2A).

**Table 3:** Sequence analysis of composite island CGI48.

Gene	Start	End	Strand	Functions
*attL*_48_	3,346,221	3,346,273	+	Left attachment site of CGI48
Sputcn32_2886	3,346,726	3,347,073	+	Hypothetical protein
Sputcn32_2887	3,347,409	3,347,561	+	Pseudo
Sputcn32_2888	3,348,921	3,347,632	−	Beta-lactamase domain protein
Sputcn32_2889	3,349,853	3,348,921	−	Hypothetical protein
Sputcn32_2890	3,350,524	3,349,859	−	Metallophosphoesterase
Sputcn32_2891	3,351,168	3,350,566	−	Conserved hypothetical protein
Sputcn32_2892	3,351,518	3,352,093	+	Hypothetical protein
Sputcn32_2893	3,352,083	3,354,299	+	Hypothetical protein
Sputcn32_2894	3,354,292	3,357,321	+	Phage integrase
Sputcn32_2895	3,357,533	3,358,846	+	Conserved hypothetical protein
Sputcn32_2896	3,359,598	3,359,224	−	Conserved hypothetical protein
Int_48_, Sputcn32_2897	3,361,269	3,360,109	−	Phage integrase
Sputcn32_2898	3,361,484	3,361,278	−	Transcription-repair coupling factor (superfamily II helicase)
Sputcn32_2899	3,361,613	3,361,819	+	Predicted transcriptional regulator, Cro/CI family
*attL*_21_	3,361,829	3,361,837	+	Left attachment site of GI21
Sputcn32_2900^a^	3,362,021	3,363,319	+	Phage Integrase
Sputcn32_2901^a^	3,363,329	3,364,162	+	Hypothetical protein
Xis_21_, Sputcn32_2902^a^	3,364,278	3,364,487	+	AlpA family phage transcriptional regulator
Sputcn32_2903^a^	3,364,910	3,365,845	+	Hypothetical protein
Sputcn32_2904^a^	3,366,023	3,366,676		Conserved hypothetical protein
Sputcn32_2905^a^	3,367,222	3,366,839	−	Hypothetical protein
Sputcn32_2906^a^	3,367,382	3,367,798	+	Putative DNA-binding protein
Sputcn32_2907^a^	3,367,890	3,368,189	+	Protein of unknown function UPF0150
Sputcn32_2908^a^	3,368,533	3,370,104	+	Type I restriction-modification system, M subunit, N-6 DNA methylase
Sputcn32_2909^a^	3,370,094	3,371,416	+	Type I restriction-modification system, specificity subunit S (EC 3.1.21.3)
Sputcn32_2910^a^	3,371,431	3,374,133	+	ATPase associated with various cellular activities, AAA_5″
Sputcn32_2911^a^	3,374,133	3,375,440	+	Conserved hypothetical protein
Sputcn32_2912^a^	3,375,839	3,378,976	+	Type I restriction-modification system, restriction subunit R (EC 3.1.21.3)
Sputcn32_2913^a^	3,379,461	3,379,039	−	Transcriptional regulator, XRE family
Sputcn32_2914^a^	3,379,625	3,380,281	+	Conserved hypothetical protein
HipB, Sputcn32_2915^a^	3,380,923	3,380,465	−	Transcriptional regulator, XRE family
HipA, Sputcn32_2916^a^	3,382,266	3,380,920	−	HipA domain protein
*attR*_21_	3,382,740	3,382,748	+	Right attachment site of GI21
Sputcn32_2917	3,383,263	3,383,625	+	Conserved hypothetical protein
Sputcn32_2918	3,384,571	3,383,654	−	Transposase, IS4 family
Sputcn32_2919	3,385,229	3,384,696	−	Conserved hypothetical protein
Sputcn32_2,920	3,386,826	3,385,240	−	Von Willebrand factor, type A
Sputcn32_2921	3,388,288	3,386,819	−	ATPase associated with various cellular activities, AAA_5
Sputcn32_2922	3,389,920	3,388,445	−	Sigma54 specific transcriptional regulator, Fis family
Sputcn32_2923	3,390,290	3,390,066	−	Hypothetical protein
Sputcn32_2924	3,390,847	3,390,707	−	Pseudo
Sputcn32_2925	3,392,615	3,390,858	−	Methyltransferase type 11
Sputcn32_2926	3,393,959	3,393,004	−	Pseudo
Sputcn32_2927	3,394,306	3,394,382	+	tRNA-Trp
*attR*_48_	3,394,303	3,394,355		Right attachment site of CGI48

a The genes in GI21.

Excision of GI followed by formation of circular forms of GI is prequisite for its horizontal transfer. Integrase is essential for GI excision and integration, and some GIs also encode recombination directionality factors (or excisionases Xis) directing the reaction toward excision (Lewis and Hatfull, 2001). We wondered whether GI21 can be excised from the CGI48 genome by recombining the attachment *attL*_21_ and *attR*_21,_ and producing *attB*_21_ and *attP*_21_ sites (Figure 2B). Quantitative PCR (qPCR) was used to quantify the excision rate by measuring the percentage of cells in the culture containing *attB*_21_, which is only present after GI21 excision. In this assay, the amount of *attB*_21_ sites is compared to the amount of the reference gene *gyrB*, which is used to quantify the total number of cells in the culture. Excisionase Sputcn32_2902 (Xis_21_) was induced in strain CN32 with 1 mM IPTG for 6 h. Additionally, the excisionases Xis_PO1_ and Xis_ANA3_ were also overexpressed in W3-18-1 and ANA-3 as a control. The results showed that Xis_21_ mediated GI21 excision, resulting in a 440-fold increase in the excision rate of GI21 and reaching (3.8 ± 0.3) × 10^−4^. However, the excision rate of GI*Spu*PO1 and GI*Ssp*ANA3 reached 17.9%–55.6% when Xis_PO1_ and Xis_ANA3_ were overexpressed, which was much higher than that of GI21 (Figure 2C). qPCR was also used to quantified the circular form of GI21 by measuring *attP*_21_, which is present after GI21 is circularized or replicated after excision. The number of *attP*_21_ is less than *attB*_21_, suggesting that GI21 is non-replicable in wild-type CN32 or expressing Xis_21_ (Figure 2D). PCR sequencing showed that the attachment sites of GI*Spu*PO1 and GI*Ssp*ANA3 were 21 bp in length, and the attachment sites of GI21 were 9 bp (Figure 2E). In CGI48, GI21 was integrated in the untranslated region between Sputcn32_2899 and Sputcn32_2917, which encoded a predicted transcriptional regulator of the Cro/CI family and a conserved hypothetical protein, respectively (Figure 2A). The excision and integration of GI21 did not cause any sequence changes in the neighboring genes. The results suggested that GI21 can be excised from CN32 by site-specific recombination of *attL*_21_ and *attR*_21_, and the shorter attachment sites may greatly limit the recombination efficiency.

**Figure 3:**
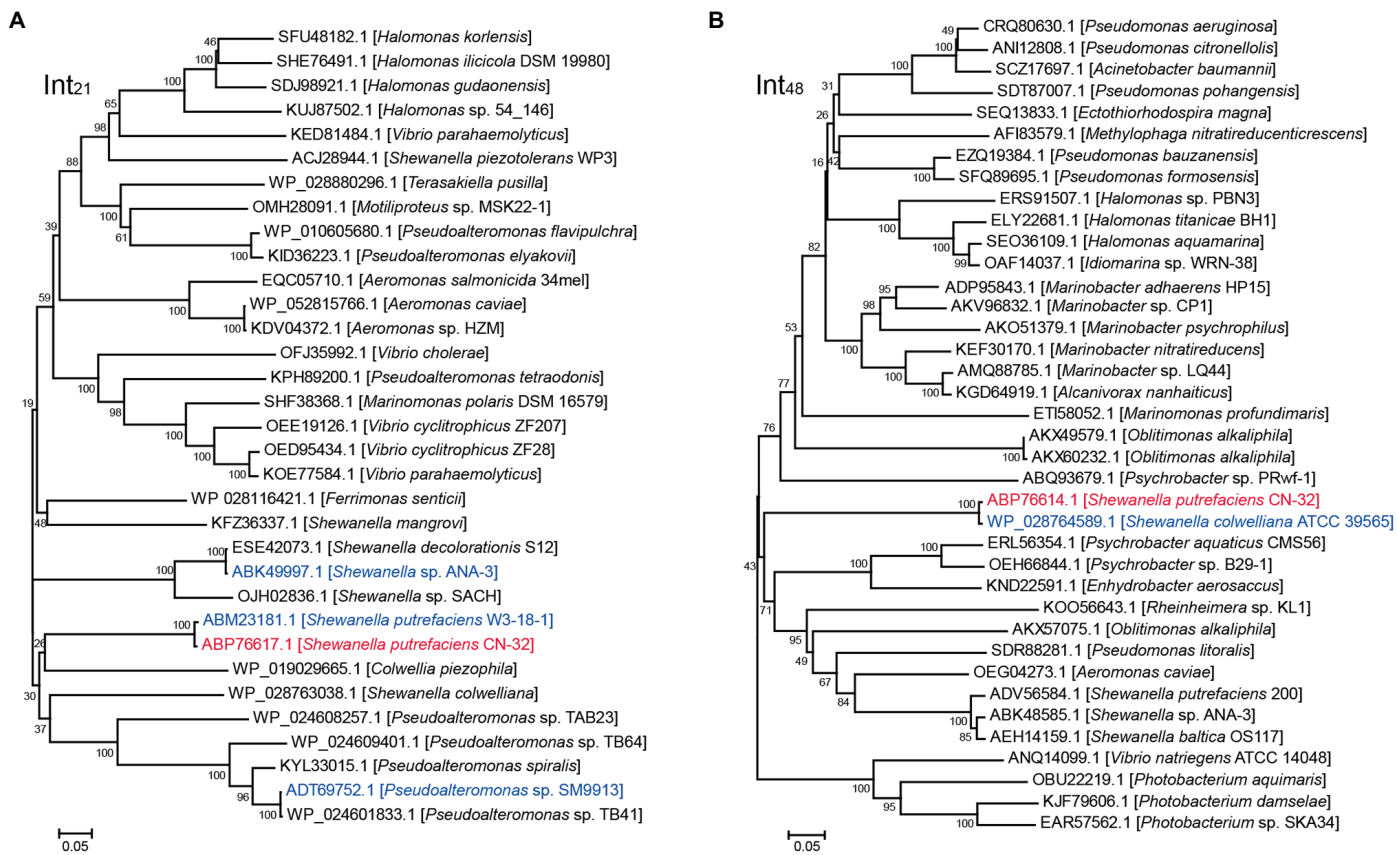
Phylogenetic analysis of Int_21_ and Int_48_ homologs. **(A)** Neighbor-joining phylogenetic tree of 32 homologs of Int_21_, and **(B)** 37 homologs of Int_48_ based on amino acid (aa) sequences. Proteins with homology to Int_21_ and Int_48_ were selected by BLASTp at the NCBI server. CN32 was indicated in red, and other genomic islands shown in Figure 2A were indicated in blue.

We then evaluated the excision of CGI48 (Figure 2B), and the integrase genes *Sputcn32_2894* and *Sputcn32_2897* were cloned into pHGECm for their overexpression. Overproduction of Sputcn32_2897 (named Int_48_) resulted in a 1.070-fold increase in the excision rate of CGI48 and reached (4.7 ± 0.6) × 10^−4^, and Sputcn32_2894 did not affect the excision of CGI48 (Figure 2F). Quantification of *attP*_48_ indicated that CGI48 is non-replicable in wild-type CN32 or expressing Int_48_ (Figure 2G). Sequence analysis showed that CGI48 was integrated in the 5′ end of tRNA^Trp^, a conserved integration locus of GIs, such as the GI in *S. colwelliana* ATCC 39565 (Figure 2A). PCR sequencing confirmed that CGI48 and GI in *S. colwelliana* ATCC 39565 shared 100% identical attachment sites of 50 bp in length, and the excision and integration did not cause sequence changes in tRNA^Trp^ (Figure 2H). Phylogenetic tree analysis of Int21 and Int48 revealed that GI21 homologs are widely distributed in *Shewanella*, *Pseudoalteromonas*, *Halomonas*, and *Vibrio* strains (Figure 3A), and CGI48 homologs are widely distributed in *Shewanella*, *Pseudomonas*, *Halomonas*, and *Photobacterium* strains (Figure 3B). Collectively, CGI48 and the component GI21 can be excised from the CN32 genome, suggesting that CGI48 is an active composite island in host bacteria.

### GI21 Encodes a HipAB Toxin–Antitoxin System

**Figure 4:**
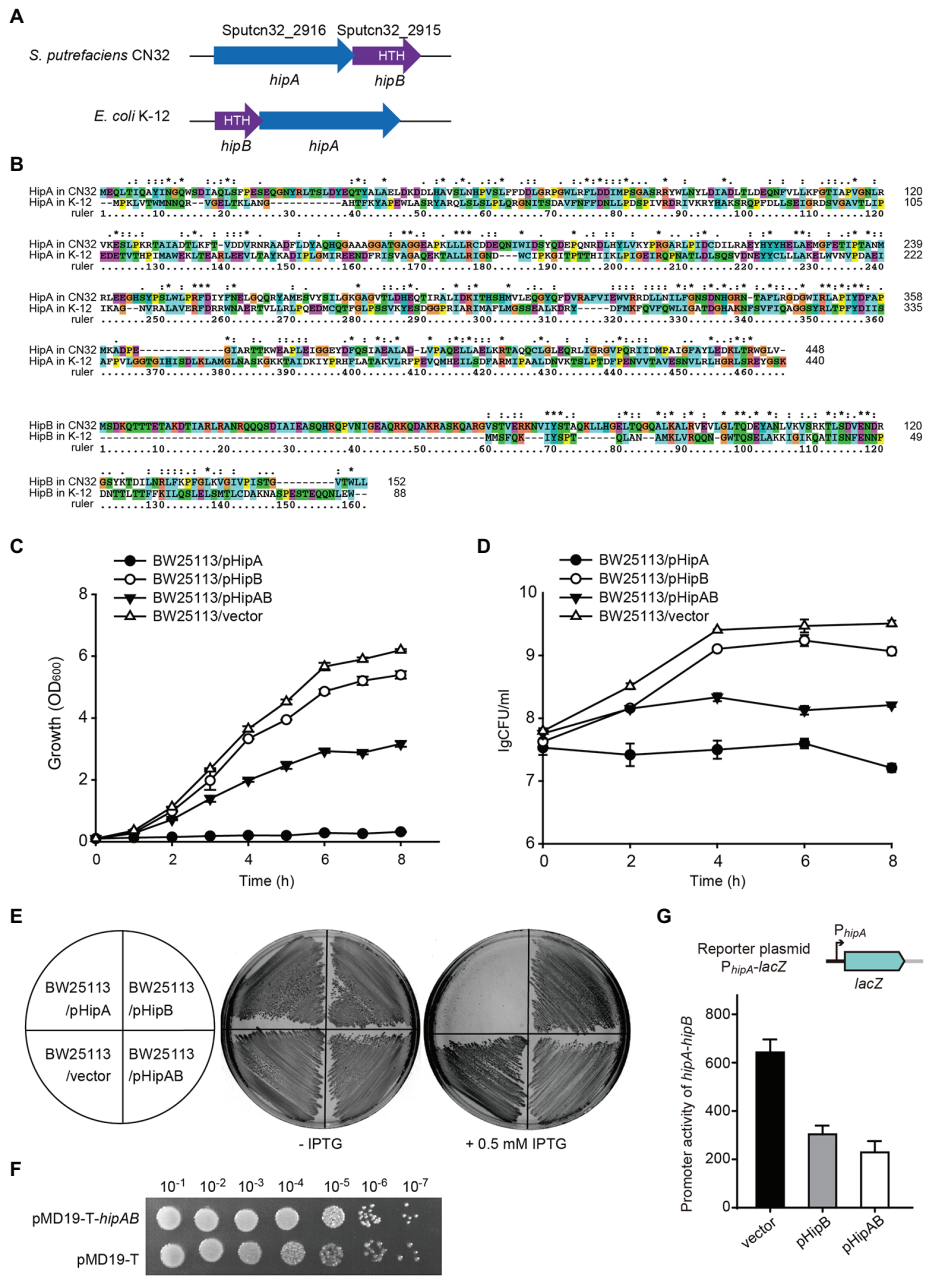
HipA and HipB in GI21 constitute a Toxin–antitoxin (TA) pair. **(A)** Comparison of the *hipA-hipB* operon in GI21 and *hipB-hipA* operon in *E. coli* K-12. **(B)** Sequence alignment was carried out using ClustalX to compare the amino acid sequence identity of HipA/HipB in *S. putrefaciens* CN32 and *E. coli* K-12. Cell growth **(C)** and cell viability **(D)** of cells overexpressing *hipA*, *hipB*, and *hipA*-*hipB via* pCA24N-based plasmids in *E. coli* BW25113. **(E)** Growth of BW25113 cells overexpressing *hipA*, *hipB*, and *hipA*-*hipB via* pCA24N-based plasmids on LB plates with and without 0.5 mM isopropyl-*β*-D-thiogalactopyranoside (IPTG). **(F)** CFU of strain BW25113 containing pMD19-T-*hipAB* or empty vector pMD19-T on LB plates with ampicillin. **(G)**The activity of the *hipA-hipB* promoter in GI21 was measured by overexpressing *hipB* or *hipA-hipB*.

In GI21, two neighboring genes that are only 4 bp apart, *Sputcn32_2916* and *Sputcn32_2915*, were identified as a putative *hipA*-*hipB* TA pair. In HipA/HipB TA system characterized in *E. coli* K-12, HipA_K-12_ toxin functions as a serine/threonine protein kinase that inhibits cell growth, and HipB_K-12_ antitoxin encoded by the gene upstream to *hipA* blocks its effects (Germain et al., 2013). Here, the putative *hipA*-*hipB* TA pair in GI21 has a genetic architecture reversed to that of *hipB-HipA* in *E. coli* K-12 (Figure 4A). *Sputcn32_2916* encodes a HipA domain protein that is 448 aa in length, and it has 40% identity and 6% coverage with HipA_K-12_. *Sputcn32_2915* encodes a XRE family transcriptional regulator of 152 aa that contains a Helix-turn-helix (HTH) domain in the C-terminal and has 33% amino acid sequence identity and 23% coverage with HipB_K-12_ (Figure 4B)_._ To determine whether *Sputcn32_2916* and *Sputcn32_2915* constitute a functional TA pair, open reading frames of the two genes were cloned into plasmid pCA24N to obtain pHipA and pHipB, respectively. Expression of *hipA* or *hipB* was induced in *E. coli* BW25113 with 0.5 mM IPTG. Cell growth (turbidity) and cell viability (CFU ml^−1^) were measured for 8 h. Overproducing HipA in BW25113 cells led to growth inhibition (Figures 4C–E). To further assess whether HipB can block the toxicity of HipA, we cloned the coding regions of *hipA* and *hipB* into plasmid pCA24N to construct pHipAB. Coexpression of *hipA* and *hipB via* plasmid pHipAB in BW25113 cells showed that HipB could partially neutralize the toxic effect of HipA (Figures 4C–E); this may result from the too high load of toxins driven by the strong *lac* promoter on the high copy number plasmid pCA24N. Then, we cloned *hipA-hipB* with its native promoter into pMD19-T to generate pMD19-T-*hipAB*. The strain BW25113/pMD19-T-*hipAB* exhibited similar cell viability with that of BW25113/pMD19-T, suggesting that HipB could fully neutralize the toxic effect of HipA under the native promoter (Figure 4F). Taken together, HipA and HipB in GI21 form a TA pair in which HipA is a potent toxin and HipB is the cognate antitoxin.

In HipA_K-12_/HipB_K-12_, the antitoxin HipB_K-12_ or the TA complex bind DNA and autoregulate the transcription of the TA operon (Black et al., 1994). Similar to HipB_K-12_, HipB in CN32 also contains a HTH domain, thus we wondered whether HipB in GI21 can regulate the *hipA-hipB* operon. Using the plasmid by fusing *lacZ* with the *hipA-hipB* promoter as the reporter, we found that overproduction of HipB exhibited 2.1 ± 0.1-fold decrease in the promoter activity compared to empty vector. Moreover, overproduction of HipA/HipB complex *via* pHipAB showed a 2.9 ± 0.4-fold decrease in the promoter activity (Figure 4G). These results suggested that GI21-encoded HipB and the HipA/HipB complex can repress the TA operon.

### GI21-Encode HipAB Stabilizes CGI48

**Figure 5:**
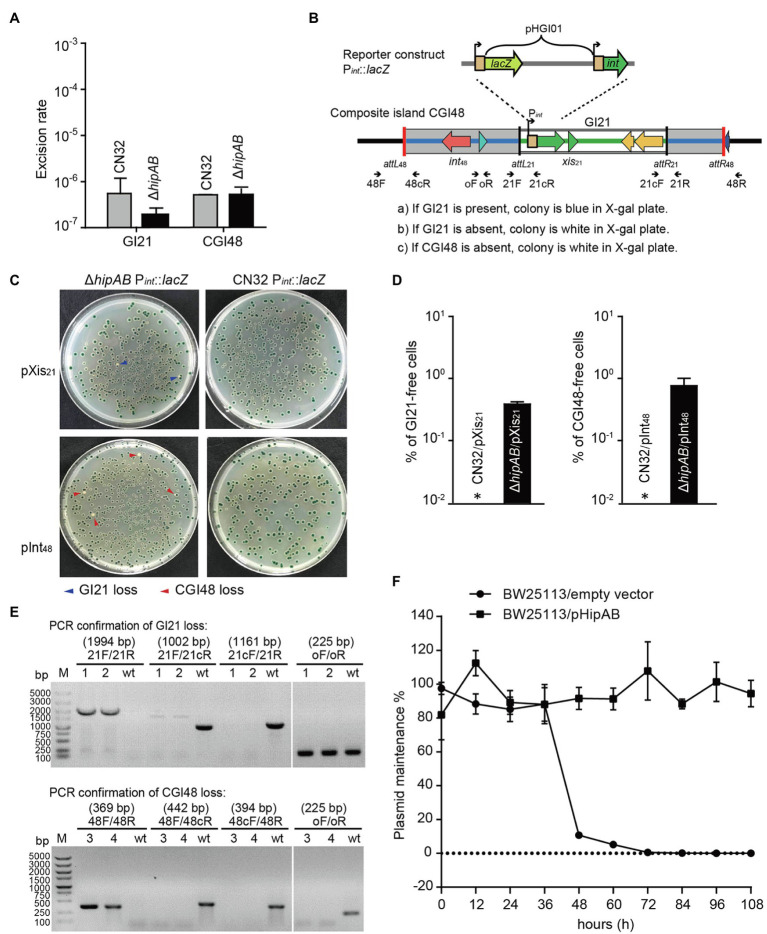
GI21-encoded HipAB promotes the maintenance of CGI48. **(A)** The excision rate of GI21 and CGI48 in the CN32 wild-type and Δ*hipAB* mutant strains. **(B)** Schematic of the *lacZ* reporter constructs in the CN32 wild-type and Δ*hipAB* strains. **(C)** Observation of GI21 loss when Xis_21_ is overexpressed (upper plates) and of CGI48 loss when Int_48_ is overexpressed (lower plates) on X-gal plates using the *lacZ* reporter system. **(D)** % of GI21-free cells (left panel) and % CGI48-free cells (right panel) were quantified by counting five plates, a representative image as shown in **(C)**. Asterisks indicate that the frequency of GI21 and CGI48 loss was below the limit of detection of the assays (<1 × 10^−5^). **(E)** Confirmation of GI21 (upper panel) and CGI48 (lower panel) loss by PCR using the indicated primers in **(B)**. 1 and 2 indicate the DNA templates extracted from the colonies with blue arrows in **(C)**; 3 and 4 indicate the DNA templates extracted from the colonies with red arrows in **(C)**; wt indicates the DNA templates from wild-type CN32 used as a control. Lane M indicates DNA Marker DL5k. The expected product sizes are indicated at the top of the primer sets. **(F)** GI21-encoded HipAB confers plasmid stability in *E. coli*. *E. coli* BW25113 harboring plasmids pHipAB and empty vector pCA24N were used in this assay. Three independent cultures were conducted, and the data are shown as means ± SDs.

To test whether the HipA/HipB TA system affects the excision of CGI48, we deleted the *hipAB* region in CN32. qPCR assays showed no significant difference in the excision rate of CGI48 in the *hipAB* deletion mutant compared to wild-type CN32 (Figure 5A). As reported in our previous study, the TA system in prophage CP4So in *S. oneidensis* stabilizes CP4So after its excision (Yao et al., 2018). We wondered whether GI21-encoding HipA/HipB played a role in the maintenance of CGI48 after its excision. A blue–white reporter screening assay was designed to detect the loss of GI21 and CGI48 after their excision. In brief, the *lacZ* gene was fused with the promoter of the integrase gene *Sputcn32_2900* to generate a P*_int_*::*lacZ* fusion and cloned into the integrative plasmid pHGI01, generating pHGI01-P*_int_*. The constructed plasmid was site specifically integrated into GI21 in CN32. Blue colonies indicated the presence of GI21 in CN32, irrespective of whether it was integrated in the host chromosome or existed in a circular form after GI21 or CGI48 was excised. White colonies indicated a complete loss of GI21 from CGI48 or a complete loss of CGI48 from the CN32 genome (Figure 5B). To activate the excision of GI21 and CGI48, Xis_21_ and Int_48_ were induced with 1 mM IPTG for 6 h, and cells were then plated on X-gal plates to detect GI21- and CGI48-free cells using the reporter plasmid (Figure 5C). No loss of GI21 was detected in wild-type CN32, and 0.39% of GI21-free cells were exhibited in the *hipAB* deletion mutant when Xis_21_ was overexpressed. Similarly, no loss of CGI48 was detected in wild-type CN32, and 0.82% of CGI48-free cells was exhibited in the *hipAB* deletion mutant when Int48 was overexpressed (Figure 5D). Then, two white colonies (indicated with blue arrows) from the Xis_21_-induced plates and two (indicated with blue arrows) from the Int_48_-induced plates were randomly selected to confirm the loss of GI21 and CGI48 (Figure 5E) by PCR. In addition, we also test the contribution of GI21-encoded HipA/HipB on plasmid stability. As shown in Figure 5F, plasmid pCA24N was completely lost from *E. coli* BW25113 after 72 h, while pHipAB which contains *hipAB* in pCA24N was stably maintained in *E. coli* after 108 h of culturing. Altogether, these results thus demonstrate that HipA/HipB not only stabilizes GI21 and CGI48 but also provides plasmid stabilization.

## Conclusion and Discussion

In this study, a new composite island, CGI48, was detected in the genome of *S. putrefaciens* CN-32. CGI48 harbors genes encoding adaptive traits, such as antibiotics and restriction–modification systems. CGI48 evolved by inserting a genomic island, GI21, showing high identity with GIs integrated in the *yicC* locus. Because the conserved *yicC* locus is intact and available in CN32 genome, GI21 might integrated into CN32 accompanied by the composite CGI48. Another possibility is that GI21 is integrated into the secondary attachment site within CGI48 genome after horizontal gene transfer. Many genomic islands preferentially integrated into a primary attachment site in the bacterial genome. Studies on the ICEs, ICE*Bs*1 found that ICE*Bs*1 can also integrate into secondary attachment site, especially when the primary site is absent. However, the excision of ICE*Bs*1 from secondary sites is greatly reduced compared to the primary site, limiting the dissemination of ICE*Bs*1 (Menard and Grossman, 2013). *In vitro* assays showed that the efficiency of integrase-mediated site-specific recombination is related to the length of the attachment site, and the reduction of the core attachment site produced a dramatically decrease in the recombination activity (Ghosh et al., 2003). Thus, we speculated that the shorter attachment sites flanking GI21 may limit its excision and stabilize the composite structure. Some composite GIs are also found to be stabilized by truncated attachment sites or integrases (Bellanger et al., 2014). In this study, we also found that a functional TA system maintain the stability of the composite GI. All these mechanisms explain the complexity and diversity of GIs.

## Data Availability Statement

The datasets presented in this study can be found in online repositories. The names of the repository/repositories and accession number(s) can be found at: https://www.ncbi.nlm.nih.gov/genbank/, CP000503; https://www.ncbi.nlm.nih.gov/genbank/, CP000681.

## Author Contributions

XW and PW conceptualized and designed the project. YZ, WW, JY, XW, DL, and PW did the investigation, data curation, and analysis. YZ, XW, DL, and PW wrote, reviewed, and edited the original draft. All authors contributed to the article and approved the submitted version.

## Funding

This work was supported by the Guangdong Major Project of Basic and Applied Basic Research (2019B030302004), the Natural Science Foundation of Guangdong Province (2019A1515011912), the Science and Technology Planning Project of Guangzhou (202002030493), Hainan Provincial Joint Project of Sanya Yazhou Bay Science and Technology City (320LH047), the Youth Innovation Promotion Association CAS (2021345 to PW), the Key Special Project for Introduced Talents Team of Southern Marine Science and Engineering Guangdong Laboratory (Guangzhou; GML2019ZD0407), the Natural Science Foundation of Hebei Province (C2019205044), Research Fund of Hebei Normal University (L2016Z03), and Science and Technology Research Project of Hebei University (ZD2018070).

## Conflict of Interest

The authors declare that the research was conducted in the absence of any commercial or financial relationships that could be construed as a potential conflict of interest.

## Publisher’s Note

All claims expressed in this article are solely those of the authors and do not necessarily represent those of their affiliated organizations, or those of the publisher, the editors and the reviewers. Any product that may be evaluated in this article, or claim that may be made by its manufacturer, is not guaranteed or endorsed by the publisher.
